# Extra-Uterine Fœtation

**Published:** 1868-06-01

**Authors:** L. T. Strother

**Affiliations:** Dayton, Indiana


### EXTRA-UTERINE FŒTATION.

BY L. T. STROTHER, M.D., DAYTON, INDIANA.

I SUBMIT the following statement of facts, which have recently come under the observation of myself and others, in relation to a very extraordinary case of extra-uterine fœtation, as it occurred in the case of Mrs. P., aged thirty-five, the mother of four children, the youngest of which is seven years old.

On the 5th of February, 1868, my worthy preceptor and friend, Dr. D. H. Crouse, was called to attend Mrs. P. in labor. The doctor obeyed the summons, supposing it nothing more than an ordinary case of accouchement. Upon his arrival, he found her experiencing pains marked by distinct intermittance, closely resembling the contractions of the uterus; but, on making an examination per vaginam, it was with very great difficulty the os tincæ could be found, as a roundish and soft mass on the left, and posteriorily, pressed the left wall of the vagina over against the right, so contracting and diminishing the vagina as to almost exclude the canal. The os was found to be slightly enlarged, with no shortening of the neck, no dilatation or softening. Upon passing his hands over the abdomen, he found the parietes to correspond to their state in a woman far advanced in pregnancy, but he immediately discovered a strange anomalism of nature—viz., two distinct tumors occupying the abdominal cavity, one quite small and hard, on the right of the linea alba, and pressed down in the right inguinal region; on the left he found a large growth filling up the whole of that side of the abdomen, which felt hard on pressure. His attention was immediately called to the small tumor in the right side in his examination, by feeling it contract through the abdominal walls during each pain.

Consultation was requested, and Dr. M. Baker, of Stockwell, was sent for. Upon his arrival, a careful examination of the case was made. He found every thing to concur as described above in every particular. They then came to the following conclusions: that, in the common acceptation of the term, she was not pregnant; the small tumor on the right side, which they could feel contract, was the uterus, that it contained nothing—tumor, polypus, or fœtus; the large growth on the left side was evidently independent of the uterus, and whatever the character of the enlargement might be, it was extra-uterine. The uterine region gave a dull sound on percussion, and there were distinct signs of fluctuation in the lower portion of the abdomen.

They then obtained from her the following history: Early in June of last year she commenced "losing blood," attended with much pain in the left side; was very much reduced and weakened by the loss of blood. A physician was called, who addressed himself to the rational treatment of the case, tonics and iron. Afterward she came under the care of different practitioners; one of which examined, pronounced and treated the case for polypoid tumor within the uterus. A "water doctor" in the meantime was called. He looked at the urine, examined her with the "touch" and speculum, and declared her pregnant. She had experienced certain signs and symptoms to lead her to suppose she was pregnant, and at this time she believed herself to be so. She did not feel at any time as during her former pregnancies; had occasional discharges at irregular intervals from the vagina during the whole period; pain was constant, and became greater as her size increased; bowels obstinately constipated all the time; breasts contained milk for some time. The doctors informed her she would not be delivered of a child; her case was a hopeless one, she could expect nothing but death, and that soon. She was very much emaciated, and at that late hour it would be impossible for her to survive an operation. They prescribed some anodyne to be taken at intervals, promising to give her all the relief in their power while she lasted. They were confident, and so expressed to her husband that her disease was either ovarian or extra-uterine pregnancy, and if the latter, the child was now dead. From that day to the day of her dissolution she had almost incessant vomiting, and complained of a burning pain at the epigastrium.

In the afternoon of the Wednesday following, February 12th, while reclining in an arm chair, she suddenly felt something give way, which was speedily followed by depression. A physician was called, who found a very slight discharge from the vagina, of a bloody, dark appearance. She was sinking fast, and he was of the opinion that she would not outlive the night. She died at nine o'clock that evening. A *post mortem* was immediately requested, but the husband would not give his consent, till, on Friday the 14th, thirty-eight hours after death, Drs. Baker, Crouse, Washburn, and myself were present; Dr. Baker conducting the autopsy, which was not held till after the funeral had been preached, and the people waiting outside presented obstacles that materially interfered to prevent a sufficiently minute and thorough examination of the *post-mortem* appearances from being obtained. A section was made in the median line, extending from the ensiform cartilage to the pubes, also a transverse incision was made an inch below the umbilicus; the integument and walls of the abdomen were carefully dissected and reflected; no marks of inflammation found. When the peritoneum was opened, we found the cavity filled with a fluid of a dark grumous appearance. The uterus was found pressed down, and over on the right side it was slightly hypertrophied, contained a small clot of blood; its walls were not thickened worthy to mention; the lining membrane was not congested. The os uteri was undilated, was not shortened; the right Fallopian tube and ovary were normal in character. We found a large tumor enveloped in a cyst occupying nearly the whole of the abdomen, and attached to almost all of the abdominal viscera. The omentum and a trace of the spleen formed the anterior coverings of the cyst; they were so strongly attached to it, and changed in appearance, that at first we did not recognize them. There were extensive adhesions existing between the appendix vermiformis, most of the ascending colon, and the cyst; the descending colon, loaded with fœcal matter, was enveloped in the cyst; portions of the ileum, jejunum, mesentery, part of the transverse colon and left lobe of the liver were attached by adhesions that could not be broken up to the posterior and superior part of the cyst. Upon opening the cyst, the body of a full-grown male fœtus was exposed, lying obliquely under the omentum, its head toward the right and above the umbilicus. The fœtus was large and well developed; seemed to be normal in every respect except the head, which was very large, the bones of which were softened. The funis was severed, child removed, blood sponged out of the sac. On following the cord, the placenta was found attached to the under side of the diaphragm, down over the spinal column and left kidney, the lower end resting in the left iliac fossa, very curious and abnormal in appearance, oblong in shape. The cord was of usual length, and once round the neck of the child. All present agreed, as a low figure, that the child would weigh seven pounds. No trace of the left Fallopian tube or ovary could be found, these organs having been entirely obliterated in the formation of the cyst. The spleen, as an organ, was not found.

I have presented this case, thinking it might be of interest to the profession, as such cases are rare in common practice; but this case is rendered more so in view of the attending circumstances. Here the child certainly lived to the full term, while the rule in this variety of gestation is, it seldom passes beyond the fifth month. From the fluid found in the abdomen of this woman, she evidently died of rupture of the sac and hæmorrhage. There were no marks of peritonitis. This was a well marked case of the abdominal species of extra-uterine pregnancy.

Whatever may have been advisable at an earlier period of this case, no one will say, I think, that an operation such as the case would have demanded should have been attempted, or was justifiable, under the circumstances found existing at the time, or after Drs. Crouse and Baker visited her.

---

INNOVATION.—"To play with important truths; to disturb the repose of established tenets; to subtilize objections and elude proofs, is too often the sport of youthful vanity, of which maturer experience commonly repents."

"One new change leaves always (as in building) a toothing or aptitude for another."

"It were good that men in their innovations would follow the example of Time itself, which indeed innovateth greatly, but quietly, and by degrees scarcely to be perceived."

